# Longitudinal Effects of Student-Perceived Classroom Support on Motivation – A Latent Change Model

**DOI:** 10.3389/fpsyg.2017.00417

**Published:** 2017-03-22

**Authors:** Rebecca Lazarides, Diana Raufelder

**Affiliations:** ^1^Department of Education, School Pedagogy, University of PotsdamPotsdam, Germany; ^2^Department of Education, School Pedagogy, University of GreifswaldGreifswald, Germany

**Keywords:** classroom characteristics, autonomy, competence, relatedness, motivation, latent change model, adolescence

## Abstract

This two-wave longitudinal study examined how developmental changes in students’ mastery goal orientation, academic effort, and intrinsic motivation were predicted by student-perceived support of motivational support (support for autonomy, competence, and relatedness) in secondary classrooms. The study extends previous knowledge that showed that support for motivational support in class is related to students’ intrinsic motivation as it focused on the developmental changes of a set of different motivational variables and the relations of these changes to student-perceived motivational support in class. Thus, differential classroom effects on students’ motivational development were investigated. A sample of 1088 German students was assessed in the beginning of the school year when students were in grade 8 (*Mean age* = 13.70, *SD* = 0.53, 54% girls) and again at the end of the next school year when students were in grade 9. Results of latent change models showed a tendency toward decline in mastery goal orientation and a significant decrease in academic effort from grade 8 to 9. Intrinsic motivation did not decrease significantly across time. Student-perceived support of competence in class predicted the level and change in students’ academic effort. The findings emphasized that it is beneficial to create classroom learning environments that enhance students’ perceptions of competence in class when aiming to enhance students’ academic effort in secondary school classrooms.

## Introduction

Longitudinal research has demonstrated that adolescents’ motivation declines consistently across the secondary school years, reaching its nadir in grade 9, with a slight subsequent recovery (Fredricks and Eccles, 2002; Watt, 2004). According to Eccles et al. (1993) stage–environment fit theory, this motivational decline may be due to a mismatch between the needs of adolescents and the opportunities they are afforded by their classroom learning environments. When aiming to effectively enhance adolescents’ academic success, it is therefore critical to examine which classroom characteristics contribute to an adaptive development of adolescents’ motivation. In this context, research based on self-determination theory (Deci and Ryan, 2002) has shown that student-perceived classroom support for autonomy, competence, and relatedness positively contributes to the adaptive development of students’ intrinsic motivation (e.g., Sierens et al., 2009; Jang et al., 2010). However, only few longitudinal studies investigated how student-perceived classroom characteristics are related simultaneously to the development of motivational variables that reflect the behavioral, cognitive and affective dimensions of students’ motivation in secondary school (for exceptions, see Ntoumanis et al., 2009; Wang and Holcombe, 2010; Dietrich et al., 2015).

This longitudinal study addressed this gap and examines how student-perceived support for autonomy, competence, and relatedness predicted the developmental changes in students’ mastery goal orientation, academic effort, and intrinsic motivation. The contribution of this study to current research thereby is twofold: First, the longitudinal study provides insights into the developmental change of adolescents’ motivation by investigating how changes in students’ mastery goal orientation, academic effort, and intrinsic motivation are interrelated. Second, the study extends current knowledge by examining whether and how student-perceived support for autonomy, competence, and relatedness predict these changes in adolescents’ motivation. When investigating classroom learning environments, the study focused on students’ perceptions of their classrooms as research has emphasized that socialization processes operate through individuals’ perceptions of their socializers’ behaviors (Eccles et al., 1983).

Students’ motivation refers to students’ beliefs, values and goal orientations that determine which tasks they choose and which effort they invest to stay on the task (Wentzel and Wigfield, 2009) and is thus central to their academic success. Motivation reflects cognitive dimensions, such as students’ goals and goal orientations in learning, but also involves affective dimensions, such as the enjoyment of tasks (Eccles and Wigfield, 2002). A behavioral dimension is students’ academic effort, and thus, the willingness to persist when facing task-related difficulties (Eccles and Wigfield, 1995). Based on these theoretical conceptualizations, this study examined the changes in students’ achievement goal orientations, academic effort, and intrinsic motivation.

Students’ achievement goal orientations are defined as cognitive representations that guide achievement-related behaviors (Elliot et al., 2005) and are frequently differentiated into mastery and performance goal orientations (Elliott and Dweck, 1988). A mastery goal orientation reflects a focus on learning and understanding (Pintrich et al., 2003). Performance-approach goal orientations are directed at demonstrating competence, while performance-avoidance goal orientations are directed at avoiding the demonstration of incompetence (Elliot and Harackiewicz, 1996). This study’s scope is limited to mastery goal orientation as it is particularly important for students’ adaptive academic development in students’ motivation (Chouinard et al., 2007; Lüftenegger et al., 2016) and achievement (Castejón et al., 2016; Valle et al., 2016).

Students’ academic effort refers to the diligent behavior that students show within the academic setting (Dietrich et al., 2015). It can be defined as the degree of difficulty in executing academic behavior which also refers to the impediments to perform the behavior (Bagozzi et al., 1990). Academic effort is an important index of motivation to achieve (Wentzel, 1996). Students’ academic effort (Trautwein, 2007; Hughes et al., 2008) is directly related to their performance in academic settings.

Intrinsic motivation is defined as “a natural inclination toward assimilation, mastery, spontaneous interest, and exploration” (Ryan and Deci, 2000, p. 70). Intrinsically motivated students engage in tasks for the fun or challenge associated with the task rather than because of external consequences. Intrinsic motivation (Wigfield and Guthrie, 2000) is an important predictor for students’ academic success.

Given their high importance for adolescents’ academic development, it is alarming that students’ mastery goal orientation (Anderman and Midgley, 1997), intrinsic motivation (Otis et al., 2005; Ntoumanis et al., 2009), and effort (Dietrich et al., 2015) decline substantially during high school. Thus, there is a lack of research that examines how students’ perceptions of their learning environment enhance or inhibit the developmental change of different dimensions of their motivation (e.g., Gottfried et al., 2009; Dietrich et al., 2015).

In their stage–environment fit theory, Eccles et al. (1993) describe that maladaptive changes in a cluster of classroom variables after the transition to high school contribute decisively to the motivational decline in adolescence: For example, a decline in autonomy support and in the quality of teacher–student relationships during high school negatively affects adolescents’ motivation (Eccles and Roeser, 2009). Accordingly, self-determination theory (Deci and Ryan, 2002) proposes that students have an inherent need for autonomy, competence, and relatedness and that learning environments that support the fulfillment of these needs facilitate self-determined learning and an adaptive motivational development.

Research based on self-determination theory (Reeve et al., 1999; Niemiec and Ryan, 2009) indicates that the cluster of autonomy-supportive teaching styles is broad and includes behaviors such as providing choice and acknowledging students’ perspective and feelings. Furthermore, autonomy-supportive teachers give the student opportunities to work in their own way, encourage the student’s academic effort, are responsive to the student’s questions and comments, and acknowledge the student’s perspective and experiences (Reeve et al., 1999; Reeve and Jang, 2006). This study focused on student-perceived autonomy support in terms of students’ feelings of being supported to work independently and in their own ways.

Research showed that students who perceive autonomy support in class also report high levels of intrinsic motivation (Reeve et al., 2004; Lazarides et al., 2015) and mastery goal orientation (Ciani et al., 2010). However, other studies showed that student-perceived autonomy support in class was not significantly associated with students’ academic outcomes, such as their academic effort (Liukkonen et al., 2010; Raufelder et al., 2015). A possible explanation for the mixed findings may be that the effect of student-perceived autonomy support on students’ motivation is moderated by students’ perceptions of the structure of the classroom (Sierens et al., 2009) and by students’ achievement level (Fei-Yin Ng et al., 2004). Student-perceived autonomy support, for example, has been shown to enhance students’ motivation only at high levels of structuredness in class, whereby structure reflects the teacher’s guidance during the process of problem solving (Sierens et al., 2009).

Another essential classroom-related dimension that contributes to the development of students’ motivation is students’ perception of their teachers as supporting their feelings of competence. Based on self-determination theory (Ryan and Deci, 2000), learning environments can help develop feelings of competence by providing optimal challenges and feedback on ability and by promoting freedom from demeaning evaluations or by applying classroom practices that emphasize task mastery. Student-perceived competence support is positively related to intrinsic motivation (Harackiewicz, 1989; Ntoumanis et al., 2009) and academic effort (Trautwein et al., 2009). This study focused on competence support in terms of student-perceived teachers’ ability feedback, teachers’ praise of improvement, and emphasis in class on the mastery of tasks. Competence-support through feedback, however, is also interrelated with students’ interpersonal relations to their teachers and peers (Raufelder et al., 2016a).

According to self-determination theory (Deci and Ryan, 2002), another motivation-enhancing factor in students’ learning environments is their perception of relatedness in class. Student-perceived relatedness in class is enhanced by teachers who are available to their students and who address their needs (Skinner and Belmont, 1993), but also by the facilitation of cooperation between students (Ntoumanis, 2005). Students in classrooms where they perceive relatedness in class tend to value their tasks (Wang, 2012), focus on the mastery of tasks (Patrick et al., 2007), and invest effort in learning (Sánchez et al., 2005). This study focused on relatedness in class in terms of student-perceived cooperativeness between students in class.

Taken together, a large amount of studies focused on the relations between student-perceived support for autonomy, competence and relatedness in class on single aspects of students’ motivation such as their interest and intrinsic motivation (Reeve et al., 2004; Lazarides et al., 2015), mastery goals (Patrick et al., 2007; Ciani et al., 2010) or effort (Trautwein and Lüdtke, 2009). However, a systematic longitudinal investigation of the joint effects of student-perceived classroom support on the development of cognitive, affective and behavioral dimensions of motivation is still needed. Furthermore, only few longitudinal studies have investigated the longitudinal relations between adolescents’ mastery goal orientations and the affective and cognitive dimensions of students’ motivation such as effort and enjoyment (e.g., Wentzel, 1996; Pintrich, 2000; Bong, 2004).

The present longitudinal study thus extends prior knowledge by examining the interrelations in changes in students’ mastery goal orientation, academic effort, and intrinsic motivation from grade 8 to grade 9. The study also investigates how the level and change in students’ mastery goal orientation, academic effort, and intrinsic motivation is predicted by student-perceived classroom support of autonomy, competence, and relatedness. The longitudinal study focused on middle-school students in grade 8 and grade 9 because adolescents’ motivation has been shown to decline consistently during high school (Eccles et al., 1998; Watt, 2004).

We hypothesize that students’ mastery goal orientation (Anderman and Midgley, 1997), effort (Dietrich et al., 2015), and intrinsic motivation (Otis et al., 2005) would decline substantially from grade 8 to grade 9 (Hypothesis 1). It is further expected that changes in students’ mastery goal orientation would be associated with changes in their academic effort and intrinsic motivation (Hypothesis 2). Additionally, we assume that student-perceived support of competence, autonomy, and relatedness in class in grade 8 would positively predict the level (grade 8) and change (Δ grade 9–grade 8) in students’ mastery goal orientation (Patrick et al., 2007; Ciani et al., 2010), academic effort (Trautwein et al., 2009), and intrinsic motivation (Lazarides et al., 2015) (Hypothesis 3). However, because studies have demonstrated that student-perceived autonomy support in class was not significantly associated with their effort (Liukkonen et al., 2010), we expect student-perceived autonomy support in class to have weak effects on students’ academic effort.

## Materials and Methods

### Participants

Quantitative questionnaire survey data of 1,088 students from 23 public secondary schools and 71 classrooms located in the federal state of Brandenburg, Germany formed the empirical base of this study. The data were collected in the beginning of the school year when students were in grade 8 (September 2011; *M*_age_ = 13.70, *SD* = 0.53, 54% girls) and again at the end of the next school year when students were in grade 9 (June 2013; dropout rate of 22.33%; *M*_age_ = 14.86, *SE* = 0.57, 55% girls).

### Procedure

Permission to conduct the study was obtained from the Ministry of Education, Youth and Sports of Brandenburg and from participating parents and students. Schools, parents, and students were thoroughly informed about the voluntary nature of their participation. Data collection occurred in classrooms. In each session, at least two members of the research team were present to inform about the aims of the study and to clarify questions related to the data assessment.

### Measures

For all measures, the reliability of the scales was acceptable. For those measures that were assessed at both time points, reliabilities are reported for both time points. For those measures that were assessed only at Time 1, reliabilities are reported for Time 1. The scales that were used in this study were well-established scales that were already used in other studies (e.g., Raufelder et al., 2013, 2016b).

#### Autonomy Support

Student-perceived autonomy support in class was measured by three items of a subscale developed by Prenzel et al. (1996), such as “During class, I can work independently on tasks” and “During class I can follow my own schedule when working on tasks.” Scale reliability was α = 0.741. Items were rated on a 6-point Likert scale (1 = *never*; 6 = *very often*).

#### Competence Support

Student-perceived competence support in class was measured by a subscale developed by Prenzel et al. (1996) containing six items, such as “During class, the teacher tells me what I can improve” and “During class the teacher praises me for good performance.” Scale reliability was α = 0.715. Items were rated on a 6-point Likert scale (1 = *never*; 6 = *very often*).

#### Relatedness in Class

Student-perceived relatedness in class was measured by a 4-item scale of the measure of students’ social climate developed by Saldern and Littig (1987). Example items are “Whenever students in our class face problems, they can rely on their classmates” and “Whenever a student does not know what to do, the other students in this class will help.” Scale reliability was α = 0.735. Items were rated on a 4-point Likert scale (1 = *not true at all*; 4 = *totally true*).

#### Effort

Students’ academic effort was assessed with three items of a subscale of the Achievement Motivation Questionnaire for Students (FLM) developed by Petermann and Winkel (2007), such as “If I consider a task to be boring, I will keep working on it” and “I invest a lot of time to get prepared for exams.” Scale reliability was α = 0.777 (Time 1) and α = 0.655 (Time 2). The answers ranged from 1 (*not true at all*) to 5 (*absolutely true*) on a 5-point Likert scale.

#### Mastery Goal Orientation

Students’ mastery goal orientation was measured using four items of a subscale of the SELLMO (scales to assess learning and achievement motivation) instrument developed by Spinath et al. (2002); e.g., “In school it is important for me to learn something interesting” and “In school it is important for me to be inspired to think about things.” Scale reliability was α = 0.663 (Time 1) and α = 0.700 (Time 2). Items were rated on a 4-point Likert scale (1 = *not true at all*; 4 = *totally true*).

#### Intrinsic Motivation

Students’ intrinsic motivation in school was measured with three items of a subscale of an adapted German version (Müller et al., 2007) of the Academic Self-Regulation Questionnaire (Ryan and Connell, 1989). Example items include “I work and learn for school because I enjoy learning” and “I work and learn for school because I enjoy solving school-related tasks.” Scale reliability was α = 0.806 (Time 1) and α = 0.737 (Time 2). Items were rated on a 5-point Likert scale ranging from 1 (*strongly disagree*) to 5 (*strongly agree*).

### Statistical Analyses

**FIGURE 1:**
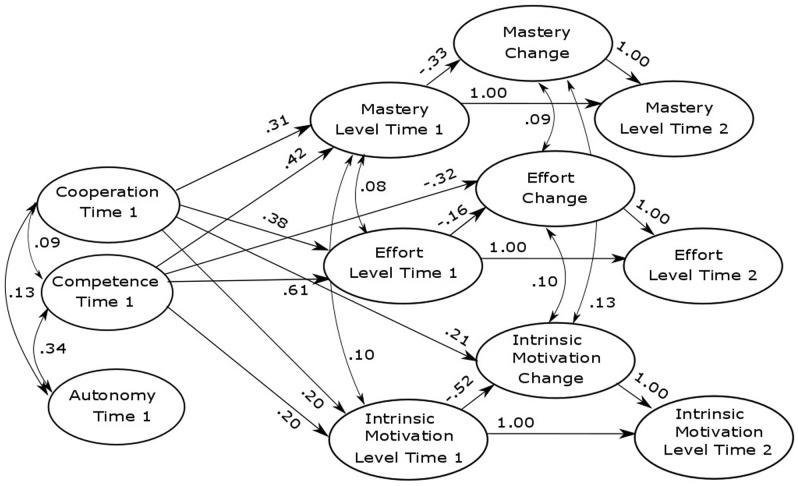
**Student-perceived classroom characteristics and students’ motivational change.** Relations between student-perceived classroom characteristics and students’ level and change in mastery goal orientation, effort, and intrinsic motivation. Unstandardized coefficients are reported. Only significant effects are depicted. Non-significant effects are reported in **Table 5**.

To test our hypotheses, we conducted a latent change model (LCM; Steyer et al., 1997; McArdle, 2009), which included three latent motivational constructs (mastery goal orientation, academic effort, and intrinsic motivation) at two measurement occasions. LCMs enable the study of interindividual differences in intraindividual change, because the true intraindividual change is modeled between two measurement points as the value of a latent variable (Steyer et al., 1997). Positive latent-change scores indicate an increase and negative scores indicate a decrease across time. As described in statistical literature (McArdle, 2009, p. 583), in order to be able to model the latent change score (Δ), we add a set of fixed values (=1) on the specific parameters between a variable value at Time 1 and at Time 2. The change score (Δ) is thereby explicitly defined. Furthermore, in order to identify the model, for each of the latent variables, the parameter between their latent change score and their level at Time 2 was set to 1 (see **Figure 1**; McArdle, 2009).

First, an unconditional LCM was conceptualized including the three latent motivational constructs. As a next step, strong measurement invariance was tested, that is, item loadings and intercepts were held equal across time points (Byrne, 1989). Measurement invariance across time is a precondition for latent change analyses (McArdle, 2009). Chi square difference testing was conducted using the scaling correction factor indicated by Satorra and Bentler (2001). Using the LCM with strong measurement invariance, we next specified the student-perceived classroom characteristics as predictors of level and change of students’ mastery goal orientation, effort, and intrinsic motivation.

The statistics software Mplus, version 7.0, was used for all analyses (Muthén and Muthén, 1998–2010), which were conducted with maximum likelihood estimation (MLR) with robust standard errors and chi-square values. To assess the reliability of the aggregated student variables, intraclass correlation coefficients (ICC) were computed for all latent variables in the model (Raudenbush and Bryk, 2002). The ICC are reported in **Table 3**. ICC values showed that the individual ratings were attributable to group membership for most of the variables. Only for students’ academic effort, ICC values indicated that 2–4% of the variance was attributable to classroom membership. This is below the critical value of ICC ≥ 0.05 which may provide evidence of a group effect (LeBreton and Senter, 2008).

We were interested in students’ intraindividual changes and their relationships to their individually perceived classroom learning environment rather than in their average motivation and the context of the classroom climate. Therefore, we decided to use the TYPE = COMPLEX function of Mplus to take the classroom nesting into account as it provides corrected standard errors and chi-square values regarding the nested structure of the data (grade 8: 1,088 students in 72 school classes; grade 9: 845 students in 67 school classes).

One case had to be excluded from the data set due to too much missing data (>80%) for the variables that were used in the analyses. Missing data were handled by using full-information maximum likelihood (FIML) estimation. Several fit criteria were employed to evaluate the goodness of fit of the models: Yuan-Bentler scaled χ^2^ (YB χ^2^, mean-adjusted test-statistic robust to non-normality), comparative fit index (CFI), root mean square error of approximation (RMSEA), and standardized root mean residual (SRMR). Hu and Bentler’s (1999) two-index strategy for determining fit was used to integrate information from the CFI and the SRMR. Models with both a CFI below 0.95 and an SRMR of greater than 0.09 are considered not to fit adequately. Other models are accepted to fit adequately. Additional, RMSEA values were used to evaluate the model fit and values below to 0.06 were considered to indicate a good fit, following Hu and Bentler (1999).

## Results

### Measurement Invariance Test

First, to test whether students’ mastery goal orientation, academic effort, and intrinsic motivation were measurement invariant across time, an unconditional LCM with no equality constraints for the latent constructs was specified (**Table 1**; step 1; configural invariance). The model fit of the unconditional LCM (**Table 1**, step 1) was acceptable: χ^2^ (155, *N* = 1087) = 434.27, *p* < 0.001, CFI = 0.93, TLI = 0.92; RMSEA = 0.04, 90% CI [0.04, 0.05], SRMR = 0.04. Second, the loadings were set invariant across time (**Table 1**; step 2; weak factorial invariance). Third, both loadings and item intercepts were set invariant across time (**Table 1**; step 3; strong factorial invariance). The model fit of the LCM, which included strong factorial invariance across time (**Table 1**, step 3), was also acceptable: χ^2^ (169, *N* = 1087) = 444.62, *p* < 0.001, CFI = 0.93, TLI = 0.92; RMSEA = 0.04, 90% CI [0.03, 0.04], SRMR = 0.04. Strong factorial invariance held for all latent constructs. In a fourth step, after testing measurement invariance for the motivational constructs, we included student-perceived support of autonomy, competence, and relatedness in class at grade 8 in the measurement model (**Table 1**, step 4). The standardized loadings from this model are reported in **Table 2**. Modification indices indicated that the model fit would substantially improve if the measurement error covariances of items 2 and 4 items and items 3 and 4 of the latent construct “student-perceived competence support” were included in the model (Byrne, 2012). After this specification, the model fit was acceptable, χ^2^ (477, *N* = 1087) = 895.97, *p* < 0.001, CFI = 0.94, TLI = 0.94; RMSEA = 0.03, 90% CI [0.03, 0.03], SRMR = 0.04 (**Table 1**, step 4a).

**Table 1:** Model fit indices for measurement invariance testing of the latent change model.

	χ^2^	*df*	Δχ^2^	Δ*df*	CFI	TLI	RMSEA	90% CI	SRMR
1	434.27	155	–	–	0.93	0.92	0.04	0.04, 0.05	0.04
2	436.77	162	4.02	7	0.93	0.92	0.04	0.04, 0.04	0.04
3	444.62	169	6.59	7	0.93	0.92	0.04	0.03, 0.04	0.04
4	1082.36	473			0.92	0.91	0.03	0.03, 0.04	0.04
4a	885.91	471			0.94	0.94	0.03	0.03, 0.03	0.04


*1 = No constraints but configural invariance; 2 = loadings invariant across time; 3 = loadings and intercepts invariant across time; 4 = measurement model including time invariance restrictions and classroom-related latent constructs; 4a = model 4 including the correlations of the measurement error variances of four items of the latent construct ‘competence support’.*

**Table 2:** Range of standardized factor loadings for latent factors.

Latent factor	Grade 8	Grade 9
Autonomy	0.41 – 0.75	
Competence	0.43 – 0.66	
Relatedness	0.41 – 0.75	
Mastery goal orientation	0.54 –0.64	0.59 – 0.66
Academic Effort	0.47 – 0.74	0.51 – 0.72
Intrinsic motivation	0.66 – 0.76	0.72 – 0.80


*Standardized latent factor loadings are reported from the final measurement model in **Table 1**, step 4a. All reported factor loadings were significant at *p* < 0.001 for standardized as well as for unstandardized estimates.*

### Descriptive Analyses

Latent means and intercorrelations, which were calculated using a confirmatory factor analysis model that included strong factorial invariance (see **Table 1**, step 3), are reported in **Table 3**. Findings showed that student-perceived support for autonomy, competence, and relatedness in class was significantly positively correlated with students’ mastery goal orientation, effort, and intrinsic motivation both at Time 1 and Time 2.

**Table 3:** Latent means, standard errors, and latent correlations.

Construct	*M* (*SE*)	ICC	2	3	4	5	6	7	8	9
(1) Mastery goal orientation grade 8	3.10 (0.03)	0.18	0.64^∗∗∗^	0.52^∗∗^	0.30^∗∗∗^	0.50^∗∗∗^	0.39^∗∗∗^	0.36^∗∗∗^	0.51^∗∗∗^	0.41^∗∗∗^
(2) Mastery goal orientation grade 9	3.06 (0.03)	0.14		0.34^∗∗∗^	0.46^∗∗∗^	0.32^∗∗∗^	0.56^∗∗∗^	0.23^∗∗∗^	0.29^∗∗∗^	0.30^∗∗∗^
(3) Academic effort grade 8	3.11 (0.04)	0.04			0.79^∗∗∗^	0.20^∗∗∗^	0.23^∗∗∗^	0.27^∗∗∗^	0.40^∗∗∗^	0.31^∗∗∗^
(4) Academic effort grade 9	3.02 (0.04)	0.02				0.09	0.22^∗∗∗^	0.21^∗∗∗^	0.24^∗∗∗^	0.24^∗∗∗^
(5) Intrinsic motivation grade 8	3.96 (0.04)	0.10					0.49^∗∗∗^	0.25^∗∗∗^	0.35^∗∗∗^	0.21^∗∗∗^
(6) Intrinsic motivation grade 9	3.96 (0.04)	0.11						0.18^∗∗∗^	0.22^∗∗∗^	0.22^∗∗∗^
(7) Autonomy grade 8	2.86 (0.04)	0.10							0.76^∗∗∗^	0.38^∗∗∗^
(8) Competence grade 8	3.96 (0.04)	0.10								0.29^∗∗∗^
(9) Relatedness grade 8	4.22 (0.04)	0.31								–


*^∗∗∗^*p* < 0.001, ^∗∗^*p* < 0.01, ^∗^*p* < 0.05.*

### Latent Change Model: Change in Mastery Goal Orientation, Academic Effort, and Intrinsic Motivation

It was expected in this study (Hypothesis 1) that students’ mastery goal orientation, academic effort, and intrinsic motivation would decrease significantly across time. This hypothesis was partially confirmed as the findings of this study showed only a tendency toward decline in mastery goal orientation (Δ *M* = -0.04, *p* = 0.087; σ Δ^2^ = 0.17, *p* < 0.001), but showed a significant decrease in students’ academic effort from grade 8 to grade 9 (Δ *M* = -0.09, *p* < 0.05; σ Δ^2^ = 0.22, *p* < 0.001). Intrinsic motivation did not decrease significantly across time (Δ *M* = 0.01, *p* = 0.977; σ Δ^2^ = 0.56, *p* < 0.001). However, the significant variance of the change pointed to substantial differences between the participants in this study regarding the developmental change in their intrinsic motivation (σ Δ^2^ = 0.56, *p* < 0.001). All three latent-change scores had significant variances, indicating significant interindividual differences in the intraindividual changes.

In this study, it was hypothesized (Hypothesis 2) that changes in students’ mastery goal orientation would be associated with changes in students’ academic effort and intrinsic motivation. Latent correlations (ϕ) showed that, as expected, the change in mastery goal orientation was positively associated with the change in effort (ϕ = 0.59, *SE* = 0.12, *p* < 0.001) and with the change in intrinsic motivation (ϕ = 0.41, *SE* = 0.08, *p* < 0.001). Additional findings showed that the change in academic effort was not significantly associated with the change in intrinsic motivation (ϕ = 0.16, *SE* = 0.10, *p* = 0.117). Furthermore, results showed that students’ average mastery goal orientation in grade 8 was significantly and negatively associated with the change in mastery goal orientation (ϕ = -0.38, *SE* = 0.06, *p* < 0.001) and academic effort (ϕ = -0.32, *SE* = 0.07, *p* < 0.001). Both mastery goal orientation and academic effort decreased over time. Thus, these findings indicated that high levels of mastery goal orientation in grade 8 inhibited the decrease in students’ mastery goal orientation and academic effort from grade 8 to grade 9.

### Student-Perceived Classroom Characteristics and Change in Motivational Orientations

To test Hypothesis 3, we modeled an LCM that included student-perceived support of autonomy, competence, and relatedness in class as predictors of the level and change in mastery goal orientation, effort, and intrinsic motivation. Standardized coefficients of this model are reported in **Table 4**. Unstandardized coefficients are reported in **Table 5**. **Figure 1** displays the significant unstandardized coefficients.

**Table 4:** Standardized coefficients from the latent change model including student-perceived classroom characteristics as predictors of the level and change of students’ motivation.

	Mastery goal orientation		Academic effort		Intrinsic motivation	
			
	Level	Change	Level	Change	Level	Change
	β (*SE*)	β (*SE*)	β (*SE*)	β (*SE*)	β (*SE*)	β (*SE*)
Level		-0.38^∗∗∗^ (0.07)		-0.24^∗∗^ (0.09)		-0.49^∗∗∗^ (0.05)
Autonomy	-0.22 (0.14)	0.06 (0.12)	-0.21 (0.11)	0.30 (0.18)	-0.11 (0.12)	-0.06 (0.12)
Competence	0.59^∗∗∗^ (0.11)	-0.10 (0.13)	0.49^∗∗∗^ (0.11)	-0.39^∗^ (0.17)	0.39^∗∗∗^ (0.11)	0.11 (0.12)
Relatedness	0.32^∗∗∗^ (0.06)	0.06 (0.07)	0.24^∗∗∗^ (0.05)	-0.02 (0.07)	0.14^∗∗^ (0.05)	0.14^∗^ (0.07)


*^∗∗∗^*p* < 0.001, ^∗∗^*p* < 0.01, ^∗^*p* < 0.05; Autonomy = student-reported support of autonomy in class; Competence = student-reported support of competence in class; Relatedness = student-reported relatedness in class.*

**Table 5:** Unstandardized coefficients from the latent change model including student-perceived classroom characteristics as predictors of the level and change of students’ motivation.

	Mastery goal orientation		Academic effort		Intrinsic motivation	
			
	Level	Change	Level	Change	Level	Change
	*B* (*SE*)	*B* (*SE*)	*B* (*SE*)	*B* (*SE*)	*B* (*SE*)	*B* (*SE*)
Level		-0.33^∗∗∗^ (.07)		-0.16^∗∗^ (0.06)		-0.52^∗∗∗^ (0.06)
Autonomy	-0.14 (0.09)	0.04 (0.07)	-0.21 (0.11)	0.20 (0.12)	-0.11 (0.12)	-0.07 (0.13)
Competence	0.46^∗∗∗^ (0.10)	-0.07 (0.09)	0.61^∗∗∗^ (0.14)	-0.32^∗^ (0.13)	0.46^∗∗∗^ (0.13)	0.13 (0.15)
Relatedness	0.31^∗∗∗^ (0.06)	0.05 (0.06)	0.38^∗∗∗^ (0.08)	-0.02 (0.07)	0.20^∗∗^ (0.07)	0.21^∗^ (0.10)


*^∗∗∗^*p* < 0.001, ^∗∗^*p* < 0.01, ^∗^*p* < 0.05; Autonomy = student-reported support of autonomy in class; Competence = student-reported support of competence in class; Relatedness = student-reported relatedness in class.*

As expected, student-perceived relatedness in class in grade 8 was significantly positively associated with students’ level of mastery goal orientation (β = 0.32, *SE* = 0.06, *p* < 0.001), academic effort (β = 0.24, *SE* = 0.05, *p* < 0.001), and intrinsic motivation in grade 8 (β = 0.14, *SE* = 0.05, *p* < 0.01). Student-perceived relatedness was furthermore significantly and positively related to the average change in students’ intrinsic motivation (β = 0.14, *SE* = 0.07, *p* < 0.05). Thus, the more the students perceived relatedness in class, the higher the increase in their intrinsic motivation.

In line with our assumptions, student-perceived competence support in grade 8 was significantly and positively related to their level of mastery goal orientation (β = 0.59, *SE* = 0.11, *p* < 0.001), academic effort (β = 0.49, *SE* = 0.11, *p* < 0.001), and intrinsic motivation in grade 8 (β = 0.39, *SE* = 0.11, *p* < 0.001). Student-perceived competence support in class in grade 8 was significantly negatively related to their change in academic effort from grade 8 to grade 9 (β = -0.39, *SE* = 0.17, *p* < 0.05). This indicated that high levels of student-perceived competence support inhibited the significant decline in students’ academic effort from grade 8 to grade 9.

In contrast to our expectations, student-perceived autonomy support was not significantly associated with students’ level of mastery goal orientation (β = -0.22, *SE* = 0.14, *p* = 0.108), academic effort (β = -0.21, *SE* = 0.11, *p* = 0.061), and intrinsic motivation in grade 8 (β = -0.11, *SE* = 0.12, *p* = 0.372). Nor with the change in their mastery goal orientation (β = 0.06, *SE* = 0.12, *p* = 0.597), academic effort (β = 0.29, *SE* = 0.18, *p* = 0.088), and intrinsic motivation (β = -0.06, *SE* = 0.12, *p* = 0.610) from grade 8 to grade 9.

The model explained significant amounts of variance in students’ level of mastery goal orientation at T1 (*R*^2^ = 0.36), academic effort (*R*^2^ = 0.22), intrinsic motivation (*R*^2^ = 0.16), and in the average change in students’ mastery goal orientation (*R*^2^ = 0.16), academic effort (*R*^2^ = 0.16), and intrinsic motivation (*R*^2^ = 0.22).

## Discussion

This longitudinal study aimed to examine the effects of student-perceived support of autonomy, competence, and relatedness in class on the level and change of students’ mastery goal orientation, academic effort, and intrinsic motivation. It contributes to current research by providing knowledge about the developmental changes of students’ mastery goal orientation, effort, and intrinsic motivation. Furthermore, it advances prior research by showing how student-perceived support of autonomy, competence, and relatedness predicted the developmental changes in different cognitive, affective and behavioral dimensions of students’ motivation.

In line with our first hypothesis (Hypothesis 1) and according to previous research (Dietrich et al., 2015), the results showed a significant change in students’ academic effort from grade 8 to grade 9 and a tendency toward decline in students’ mastery goal orientation. Previous research has shown that students’ mastery goal orientation decreased during the transition from elementary to middle school (Anderman and Midgley, 1997); thus, based on the findings of this study, we may assume that students’ mastery goal orientation declines most sharply in times of educational transitions but stabilizes during the later years of high school.

Contrasting with previous results (Gottfried et al., 2001; Otis et al., 2005), our findings showed that students’ intrinsic motivation did not decline significantly across time. A possible explanation for the non-significant findings may be that the decline in students’ intrinsic motivation has been shown to be domain-specific with mathematics showing the greatest decline (Gottfried et al., 2001). As this study focused on students’ general intrinsic motivation, the domain-specific differences in intrinsic motivation may have leveled out the declining trend of students’ intrinsic motivation.

As expected (Hypothesis 2), the findings of this study showed that a developmental change in students’ mastery goal orientation was associated with the developmental change in academic effort and intrinsic motivation across the school year. Extending current research that often focused on the developmental trends of different facets of motivation without examining their associations (e.g., Gottfried et al., 2001; Otis et al., 2005; Ntoumanis et al., 2009). Interestingly, students’ developmental change in academic effort was not significantly associated with the change in intrinsic motivation. Our findings thus point to a key role of students’ mastery goal orientation in the developmental processes of their motivation and provide further knowledge about the previously reported adaptive effects of mastery goal orientation in school (e.g., Elliot, 1999; Harackiewicz et al., 2000; Wolters, 2004). The findings may be interpreted as evidence that the pursuit of mastery goal orientation elicits not only developmental changes in affective processes, for example, in students’ enjoyment of learning (Lüftenegger et al., 2016), but also in cognitive and behavioral processes, for example, in students’ academic effort (Elliot, 1999).

Our assumption (Hypothesis 3) that student-perceived support for autonomy, competence and relatedness in class would be related to the level and change of different dimensions of students’ motivation was only partly confirmed. The findings of the study showed that only student-perceived support of competence and relatedness in class in grade 8 was significantly associated with students’ level of mastery goal orientation, effort, and intrinsic motivation in grade 8. In contrast to previous research (Ciani et al., 2010; Lazarides et al., 2015), our findings indicated that student-perceived autonomy support was not significantly associated with students’ level of mastery goal orientation in grade 8 nor with the change in their mastery goal orientation, academic effort, and intrinsic motivation from grade 8 to grade 9. A possible explanation for this finding might be the fact that the effects of student-perceived autonomy support in class on students’ motivation may depend on the structured nature of classroom teaching (Sierens et al., 2009), perceived competence (Patall et al., 2014) and on students’ achievement level (Fei-Yin Ng et al., 2004). Focusing on autonomy-supportive teaching in terms of task choice, Sierens et al. (2009), for example, showed that student-perceived autonomy support enhanced students’ cognitive engagement only at high levels of structure in class. Patall et al. (2014) furthermore demonstrated that only when students felt highly competent on a task, making choices further enhanced their motivation. However, when students felt less competent on a task, task choices diminished perceived intrinsic motivation. Such interaction effects thus may explain the non-significant effects of student-perceived autonomy support on students’ motivation in this study and future research should take into account such interdependencies when investigating the relations between student-perceived support of autonomy, competence, and relatedness and students’ motivational development in class. Another possible explanation for the non-significant effect of student-perceived autonomy support on students’ motivational development may be that this study focused on autonomy-supportive teaching in terms of students’ perceptions of possibilities to work independently and to follow their own schedule when working on school-related tasks. However, research based on self-determination theory (Reeve et al., 1999; Reeve, 2002) describes that autonomy-supportive teaching also includes other dimensions of teachers’ behaviors, such as the acknowledgment of students’ perspectives and the responsiveness to the students’ questions. Further studies are needed to examine how different dimensions of autonomy support affect the development of different facets of students’ motivation.

Interestingly, in this study, student-perceived support for competence in class primarily enhances the positive development of the behavioral dimension of students’ motivation, which was assessed by academic effort. Adolescents’ experiences of positive relationships with teachers and peers, in turn, were related to an adaptive development of students’ intrinsic motivation. This may be because competence-supportive teaching strategies may enhance students’ perceptions of their own competence rather than their enjoyment in learning, whereas high levels of perceived competence may be related to a greater effort and persistence in learning (Eccles and Wigfield, 2002).

Regarding the implications of this study for classroom instruction, the findings emphasize a differential functioning of different aspects of classroom support in the enhancement of an adaptive motivational development. While it seems to be important to implement competence-enhancing strategies when aiming to increase students’ motivation at a behavioral level, the facilitation of supportive relationships with both peers and teachers in secondary school seems to be beneficial when aiming to enhance students’ intrinsic motivation. Previous research based on stage-environment fit theory suggested that after the transition to secondary school, students often perceive their teachers as more distant and less supportive than teachers in elementary school (Roeser et al., 1993). Therefore, in our view, teachers should create a climate of supportiveness in class based on well-being and reciprocal aid (Ruzek et al., 2016). They should also adopt a teaching style that includes active feedback, which answers students’ need for competence support (Niemiec and Ryan, 2009).

### Limitations, Strengths, and Conclusions

When interpreting the current results, several limitations should be considered. First, this study was not domain-specific in its examination of either support of autonomy and competence or relatedness. Previous research (Gottfried et al., 2001) has, however, shown that the motivational development in adolescence is often domain-specific. Thus, future studies should take into account different school subjects when examining changes in students’ motivation.

Second, it would also be fruitful to differentiate between effects on the classroom level and on the individual student level because previous research has shown that students’ average motivation is predicted by teachers’ interest (Schiefele and Schaffner, 2015) and support (Dietrich et al., 2015) on the classroom level.

Third, it can be said that students’ perceived support of relatedness was based only on their peers in class. On the other hand, one strength of this study may be its examination of both student-perceived support from peers (relatedness) and teachers’ support (support for student autonomy and competence).

Fourth, in this study, only students’ self-reported perceptions of their classroom characteristics were assessed. Previous research (Gniewosz and Noack, 2012) has demonstrated that socializers’ beliefs and behaviors affect adolescents’ beliefs only if those beliefs are consciously perceived by the adolescent. However, by using only student self-reports, the examined relations between students’ perceptions of their classroom characteristics and their motivation may be biased due to shared variance attributable to the same method effect (Chan, 2009). To validate the findings of this study, future research should include teacher reports of classroom characteristics or external observer ratings (Holzberger et al., 2013; Praetorius et al., 2014). However, previous studies that focused on students’ motivation have shown that teacher and student self-reports were not highly correlated (see Skinner and Belmont, 1993; Wentzel et al., 2010) and that, compared to teacher reports, students’ reports of their classroom characteristics more strongly predicted the development of students’ motivation (Clausen, 2002).

Fifth, there are limitations in the psychometric quality of the variables that were used in this study. This instruments assessing students’ perceptions of support for autonomy, competence and relatedness in class, which proved to have good psychometric qualities when used with other populations (0.85–0.94; Prenzel et al., 1996). The same applies for the scales assessing mastery goal orientation (0.81; Schöne et al., 2004) and intrinsic motivation (0.92; Müller et al., 2007). However, the scales showed restricted psychometric qualities in the present population. Because of their substantial contribution to the model, we decided not to remove the variables.

Despite these limitations, the study has several strengths. The simultaneous examination of the predictive effects of different student-perceived classroom characteristics on different motivational aspects extends current research about the social antecedents of adolescents’ academic development in secondary school classrooms.

Furthermore, the complex statistical analyses are based on data from a large sample of adolescent students and allow a fine-grained analysis of the developmental changes in adolescents’ motivation during high school.

Overall, the findings provide an essential insight into developmental changes that occur in early adolescents’ motivation—predicted by student-perceived support of autonomy, competence, and relatedness in class.

## Conclusion

The current findings suggest that student-perceived support for competence and relatedness in secondary classrooms are essential starting points to prevent students’ motivational decline that often begins in early adolescence. The support of competence inhibits maladaptive student development and bolsters students’ effort to persist in later years of high school, whereas the support of relatedness enhances a positive developmental trend in students’ intrinsic motivation.

## Ethics Statement

This study was carried out in accordance with the recommendations of the guidelines of the Ministry of Education, Youth and Sports of Brandenburg (http://bravors.brandenburg.de/verordnungen/wissuv_1998) with written informed consent from all subjects. All subjects gave written informed consent in accordance with the Declaration of Helsinki. The protocol was approved by the Ministry of Education, Youth and Sports of Brandenburg.

## Author Contributions

All authors agree to be accountable for the content of the work. RL did the statistical analyses and wrote the main part of the paper. DR reviewed the paper, contributed decisively to the discussion part and added comments on the manuscript throughout the process of manuscript development.

## Conflict of Interest Statement

The authors declare that the research was conducted in the absence of any commercial or financial relationships that could be construed as a potential conflict of interest.
